# Burden of caregiving for cardiovascular dysautonomia in Parkinson’s disease

**DOI:** 10.1007/s10286-022-00888-9

**Published:** 2022-08-28

**Authors:** Claudia Ledda, Elisa Montanaro, Gabriele Imbalzano, Aristide Merola, Ilaria Bruno, Carlo Alberto Artusi, Maurizio Zibetti, Mario Giorgio Rizzone, Marco Bozzali, Gabriele Sobrero, Fabrizio Vallelonga, Simona Maule, Leonardo Lopiano, Alberto Romagnolo

**Affiliations:** 1 Department of Neuroscience “Rita Levi Montalcini”, University of Turin, Via Cherasco 15, 10126 Turin, Italy; 2 SC Neurologia 2U, AOU Città della Salute e della Scienza, Via Cherasco 15, 10126 Turin, Italy; 3 Department of Neurology, Wexner Medical Center, Ohio State University, Columbus, OH USA; 4 Department of Medical Sciences, Internal Medicine Division, Autonomic Unit and Hypertension Unit, University of Turin, Turin, Italy

**Keywords:** Cardiovascular autonomic neuropathy, Dysautonomia, Parkinson’s disease, Caregiver burden, Zarit Burden Interview

## Abstract

**Purpose:**

We sought to estimate the impact of cardiovascular autonomic neuropathy (cAN) on informal caregivers of patients with Parkinson’s disease (PD), defined as individuals providing regular care to a friend, partner, or family member with PD, and to evaluate the mutual relationship between caregiver burden and patient health-related quality of life (HRQoL).

**Methods:**

We enrolled 36 consecutive patients with PD and their informal caregivers. Patients underwent a detailed motor, autonomic, cognitive, and functional assessment. Caregivers were assessed using the Zarit Burden Interview (ZBI). Differences in caregiver burden, expressed by the ZBI score, and strength of association between caregiver burden, cAN, and HRQoL were assessed using analysis of covariance (ANCOVA), logistic regression, and linear regression analyses. Analyses were adjusted for patients’ age, PD duration, and motor and cognitive disability, as well as caregivers’ age.

**Results:**

Moderate-severe caregiver burden was reported in 41.7% of PDcAN^+^ versus 8.7% of PDcAN^−^ (*p* < 0.001). The ZBI score was increased in PDcAN^+^ versus PDcAN^−^ (31.5 ± 3.4 versus 15.2 ± 2.3; *p* < 0.001), with tenfold higher odds (*p* = 0.012) of moderate-severe caregiver burden in PDcAN^+^, even after adjusting for potential confounders. The ZBI score correlated with cAN severity (*p* = 0.005), global autonomic impairment (*p* = 0.012), and HRQoL impairment (*p* < 0.001).

**Conclusion:**

These results highlight the significant impact of cAN on PD caregivers and the need for targeted interventions addressing this frequently overlooked and insufficiently treated source of nonmotor disability in PD.

## Introduction

Cardiovascular autonomic neuropathy (cAN), usually characterized by orthostatic hypotension (OH) with or without supine hypertension, is one of the most common and disabling features of advanced Parkinson’s disease (PD), with an estimated prevalence of 30–50% [1–3]. Several studies have shown an association between cAN and faster motor and cognitive decline, higher rate of falls, hospitalizations, and death [2, 4–9], greater cerebral atrophy [10], and significant impact on activities of daily living (ADL) and health-related quality of life (HRQoL) [2].

Advanced PD and cAN may pose a significant burden on patients with PD and their caregivers, in particular “informal caregivers,” who are defined as nonprofessional individuals providing regular care to a friend or a family member. This proves relevant when considering that, differently from other PD symptoms, cAN symptoms can be treated with pharmacological and nonpharmacological interventions. So far, the caregiver burden due to PD-associated autonomic dysfunction has been scarcely investigated [11–13].

This study sought to evaluate the impact of cAN on the caregiver burden, independently from the patient’s cognitive status and motor disability, and the relationship between caregiver burden and patient’s HRQoL. Results are expected to assist with the development and implementation of patient- and caregiver-centered interventions for autonomic complications in advanced PD.

## Methods

A cohort of consecutive patients with PD and their respective informal caregivers were asked to participate in the study during their regular outpatient visits at the Movement Disorders Center of the “Città della Salute e della Scienza of Turin” hospital, between January 2021 and June 2021.

### Eligibility criteria

Inclusion criteria were a diagnosis of idiopathic PD, as per the Movement Disorder Society (MDS) diagnostic criteria [14], age between 18 and 80 years, and receiving care from an informal caregiver.

Exclusion criteria were diabetes mellitus or other disorders potentially associated with cAN [15], and any clinical feature preventing the quality of cAN instrumental assessment (including, but not limited to cardiac arrhythmias, severe obstructive or restrictive pulmonary disease, and major psychiatric disorders).

The local ethics committee approved the study, and all participants provided written informed consent.

### Patient assessment

#### Clinical assessment

Motor disability was assessed by means of the MDS Unified Parkinson’s Disease Rating Scale (MDS-UPDRS) part III [16]. The cognitive status was evaluated using the Montreal Cognitive Assessment (MoCA) [17], with a cutoff score for dementia < 21/30 [18].

Nonmotor and motor experience of daily living impairment were assessed by means of the MDS-UPDRS part I and II, respectively [16]; HRQoL was evaluated with the 39-item Parkinson’s Disease Questionnaire (PDQ-39, single index) [19]. The degree of global autonomic dysfunction was assessed by means of the Scales for Outcomes in Parkinson’s Disease-Autonomic questionnaire (SCOPA-AUT) [20]. Medication was logged, and the levodopa equivalent daily dose (LEDD) was calculated according to a validated conversion table [21].

#### Cardiovascular autonomic neuropathy assessment

Patients underwent a standardized battery of cardiovascular autonomic tests (DAN Test Microlab, Padua, Italy) [22], including heart rate variability (HRV) and blood pressure (BP) assessments during deep breathing, Valsalva maneuver, and lying to standing. HRV was assessed by means of a single-lead electrocardiogram, while BP was measured using a beat-to-beat monitor (Finapres; Finapres Medical Systems B.V., The Netherlands) and a manual sphygmomanometer. Autonomic tests were performed in the morning, at least 3 h after the last meal, during the patient’s OFF state (i.e., at least 12 h after the last dose of antiparkinsonian medication).

During deep breathing test, the expiratory to inspiratory ratio was calculated from the maximum and minimum R–R interval. The Valsalva maneuver (VM) consisted of requiring the subject to blow into a mouthpiece at a pressure of 40 mmHg, and hold the breath for 15 s. The ratio between the longest and the shortest R–R interval was derived. BP changes during the four phases of VM were also analyzed. The lying-to-standing test was performed by asking the subject to lie for 10 min and then stand up quickly and remain in a stable position for 5 min during which blood pressure and heart rate were measured at predefined intervals (supine position and after 1, 3, and 5 min of standing). OH was defined as a sustained reduction of systolic BP ≥ 20 mmHg or diastolic BP ≥ 10 mmHg within 3 min of standing [23].

Cardiovascular autonomic tests, adjusted for age, were scored on the basis of the Composite Autonomic System Score (CASS) [22], excluding sudomotor measures.

### Caregiver burden assessment

Informal caregivers, defined as “any person who, without being a professional or belonging to a social support network, constantly lives with the patient and, in some way, is directly implicated in his/her care, or is directly affected by the patient's health problem” [13], were asked to report the degree of their perceived distress due to patient’s illness. They were contacted during planned outpatient visits or, if not present, by phone call.

We used the Zarit Burden Interview (ZBI), a self-administered questionnaire that investigates the level of psychosocial burden resulting from the responsibility toward the relative [24]. It consists of 22 questions, rated from 0 to 4, with the total score ranging from 0 to 88; items take into consideration common areas of concern, such as health, finances, social life, and interpersonal relationships. Higher scores indicate a higher caregiver burden, with the following levels: absent-mild (score 0–20), mild-moderate (21–40), moderate-severe (41–60) and severe (61–88). This questionnaire has demonstrated validity and reliability in caregivers of individuals with chronic conditions, such as PD [13].

### Statistical analysis

Descriptive data are reported as mean ± standard deviation, or percentages, depending on their continuous or categorical distribution. Differences between patients with and without cAN (PDcAN^+^ and PDcAN^−^), and between their caregivers, were evaluated by means of the Mann-Whitney non-parametric test or Fisher’s exact test, as appropriate. The analysis of covariance (ANCOVA) was used to evaluate differences in ZBI score (dependent variable) between the two groups of caregivers (fixed factor), adjusting for patient’s age, disease duration, MoCA score, and MDS-UPDRS part III score, as well as caregiver’s age (covariates). Moreover, an ANCOVA model was used to evaluate differences in ZBI scores between male and female caregivers, and between caregivers of patients who reported falls and those who did not. ANCOVA assumption of homogeneity of variance was verified. The strength of association between cAN (independent variable) and an at least moderate-severe caregiver burden (i.e., ZBI score ≥ 41; dependent variable) was evaluated by means of a logistic regression analysis, adjusting for patient’s age, disease duration, MoCA score, and MDS-UPDRS part III score, as well as caregiver’s age. The Hosmer and Lemeshow’s goodness-of-fit test was applied. Correlations between ZBI scores and CASS, SCOPA-AUT, and PDQ-39 scores were evaluated by means of a linear regression analysis, adjusting for patients’ age, disease duration, MoCA score, MDS-UPDRS part III score, and caregivers’ age. Patients with ≥ 15% clinical/instrumental missing data were excluded from the analyses. All analyses were performed by Statistical Package for the Social Sciences (SPSS 27.0 for Macintosh, Chicago, IL), using two-tailed *p*-values with a level of significance of 0.05.

## Results

A total of 36 consecutive patients with PD (24 males and 12 females) and 36 informal caregivers (14 males and 22 females) were enrolled in the study; one patient was excluded from the analyses because of an incomplete cAN assessment.

Out of the 35 patients included in the analyses (24 males and 11 females; 68.6 and 31.4%), 12 (34.3%) were PDcAN^+^ and 23 (65.7%) were PDcAN^−^. The 35 primary caregivers (13 males and 22 females; 37.1% and 62.9%) were mainly spouses or partners (*N* = 29; 82.9%), or (to a lesser extent) sons and daughters (*N* = 6; 17.1%).

There was an association between cAN and higher impairment in nonmotor (*p* = 0.034) and motor (*p* = 0.037) experience of daily living, higher prevalence of falls (*p* = 0.038), lower MoCA scores (*p* = 0.005), and worse HRQoL (*p* = 0.001) (Table 1). Moreover, PDcAN^+^ showed a trend toward an older age, higher motor disability, and a higher rate of dementia, without reaching full statistical significance (Table 1).

**Table 1:** Patients’ and caregivers’ characteristics

	All patients (*n* = 35)	cAN− (*n* = 23)	cAN+ (*n* = 12)	*p*-Value
Gender (M/F)	24/11 (68.6%/31.4%)	15/8 (65.2%/34.8%)	9/3 (75.0%/25.0%)	0.709
Age, years	69.2 ± 8.3 (44–80)	67.6 ± 7.8 (44–79)	72.2 ± 8.7 (55–80)	0.068
Disease duration, years	8.1 ± 4.6 (4–19)	7.9 ± 4.2 (4–1)	8.4 ± 5.4 (5–18)	0.986
Age at Parkinson’s disease onset, years	61.7 ± 9.0 (37–77)	60.7 ± 8.7 (37–76)	63.7 ± 9.7 (47–77)	0.362
MDS-UPDRS-I	13.5 ± 6.8 (3–29)	12.0 ± 7.0 (3–29)	16.6 ± 5.6 (7–27)	0.034
MDS-UPDRS-II	12.8 ± 6.1 (3–24)	11.3 ± 6.1 (3–24)	15.5 ± 5.3 (7–23)	0.037
MDS-UPDRS-III	31.6 ± 13.1 (9–62)	29.6 ± 14.6 (9–62)	35.3 ± 6.9 (18–54)	0.073
Falls (yes/no)	12/23 (34.3%/65.7%)	5/18 (21.7%/78.3%)	7/5 (58.3%/41.7%)	0.038
Montreal Cognitive Assessment	23.7 ± 4.3 (14–29)	24.7 ± 4.0 (14–29)	21.7 ± 3.1 (15–25)	0.005
Dementia (yes/no)	8/27 (22.9%/77.1%)	3/20 (13.0%/87.0%)	5/7 (41.7%/58.3%)	0.091
PDQ-39 SI	31.0 ± 16.9 (7.2–69.3)	26.2 ± 16.1 (7.2–65.6)	40.1 ± 15.1 (17.2–69.3)	0.001
Composite autonomic system score	2.7 ± 2.2 (0–7)	1.4 ± 1.2 (0–4)	5.1 ± 1.4 (3–7)	0.011
SCOPA-AUT	19.9 ± 8.9 (4–46)	17.9 ± 9.1 (4–32)	21.8 ± 16.1 (15–46)	0.047
Caregivers				
Gender (M/F)	13/22 (37.1%/2.9%)	10/13 (43.5%/56.5%)	3/9 (25.0%/75.0%)	0.463
Age, years	63.7 ± 12.1 (37–81)	60.7 ± 11.9 (37–81)	69.3 ± 10.6 (49–80)	0.041
Zarit Burden Interview	20.8 ± 15.9 (153)	14.0 ± 13.5 (1–51)	33.8 ± 11.6 (18–53)	< 0.001

Results are reported as average ± standard deviation (range) or absolute value (percentage), as appropriate. *cAN* cardiovascular autonomic neuropathy, *MDS-UPDRS* Movement Disorder Society Unified Parkinson’s Disease Rating Scale, *PDQ-39 SI* 39-item Parkinson’s disease questionnaire single index, *SCOPA-AUT* Scales for Outcomes in Parkinson’s Disease-Autonomic questionnaire

### Caregiver burden

**Fig. 1:**
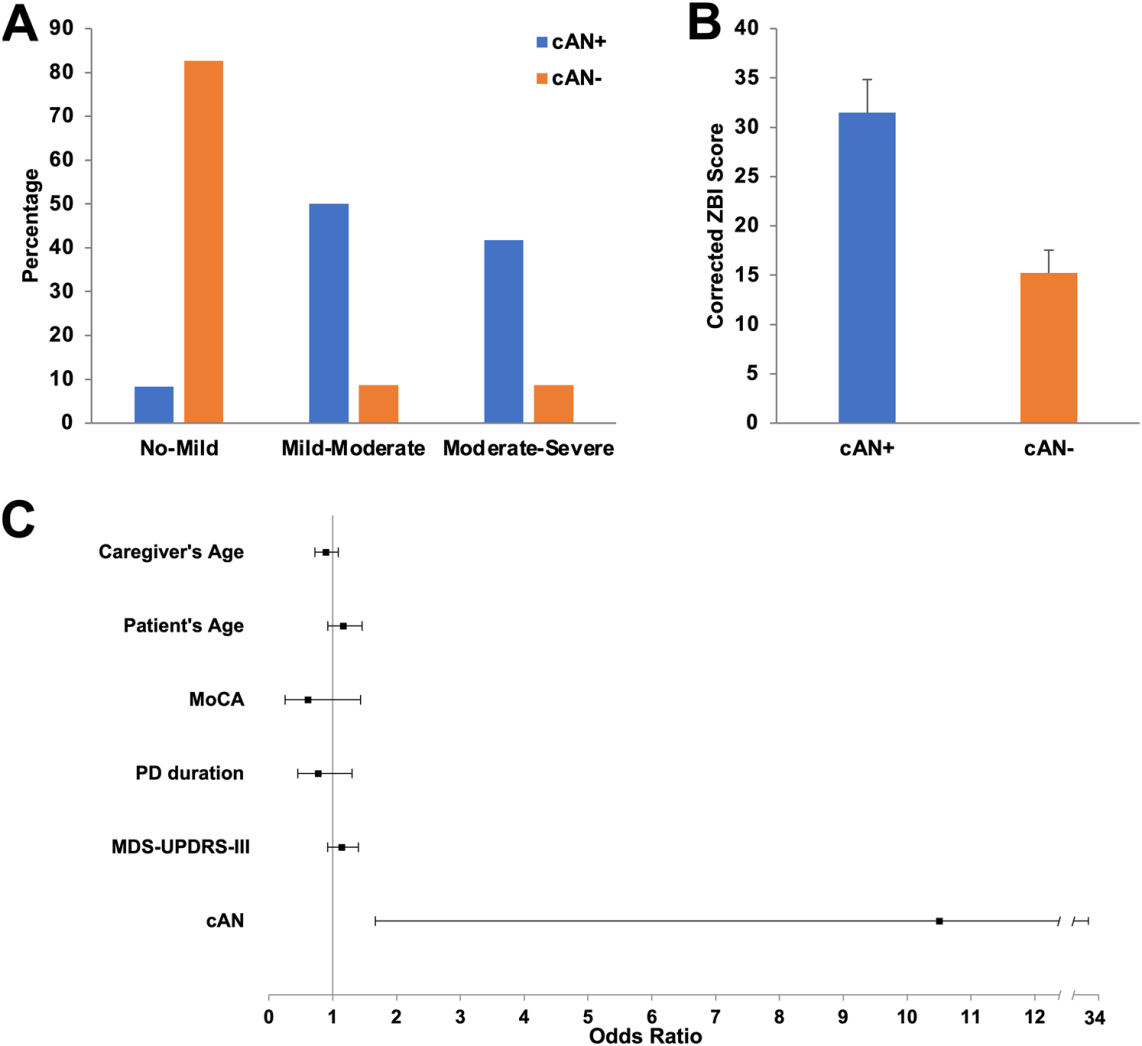
Association between cAN and higher caregiver burden. cAN is associated with a higher caregiver burden, as measured by means of the ZBI score (**A**, **B**), with a tenfold higher probability of producing a moderate-severe caregiver burden (**C**). *cAN* cardiovascular autonomic neuropathy, *ZBI* Zarit Burden Interview, *MoCA* Montreal Cognitive Assessment, *PD* Parkinson’s disease, *MDS-UPDRS* Movement Disorder Society Unified Parkinson’s Disease Rating Scale

Burden severity was significantly different between the two groups of caregivers (*p* < 0.001): no-mild burden was reported in 8.3% of PDcAN^+^ versus 82.6% of PDcAN^−^ caregivers, while moderate-severe burden was reported in 41.7% of PDcAN^+^ versus 8.7% of PDcAN^−^ (Fig. 1A). After correcting for patients’ age, disease duration, level of cognition, motor disability, and caregivers’ age, the burden of PDcAN^+^ caregivers remained significantly higher (corrected mean ± standard error: 31.5 ± 3.4 versus 15.2 ± 2.3; *F*(1, 28) = 14.013; *p* < 0.001) (Fig. 1B). No between-group differences were found in caregivers’ gender (corrected mean ± standard error: 18.2 ± 3.9 (males) versus 22.7 ± 2.9 (females); *F*(1, 28) = 0.743; *p* = 0.396).

The caregivers of patients reporting falls showed higher ZBI scores in both groups (PDcAN^−^ and PDcAN^+^). However, these differences no longer remained statistically significant after correcting for patients’ age, disease duration, cognition, motor disability, and caregivers’ age (PDcAN^−^ group: 21.4 ± 6.7 versus 12.4 ± 2.8, *p* = 0.206; PDcAN^+^ group: 38.4 ± 4.9 versus 29.1 ± 6.0, *p* = 0.310).

The logistic regression analysis showed that cAN was independently associated with tenfold higher odds of producing a moderate-severe caregiver burden [odds ratio (OR) 10.500; 95% confidence interval (CI) 1.668–33.590; *p* = 0.012] (Fig. 1C).

**Fig. 2:**
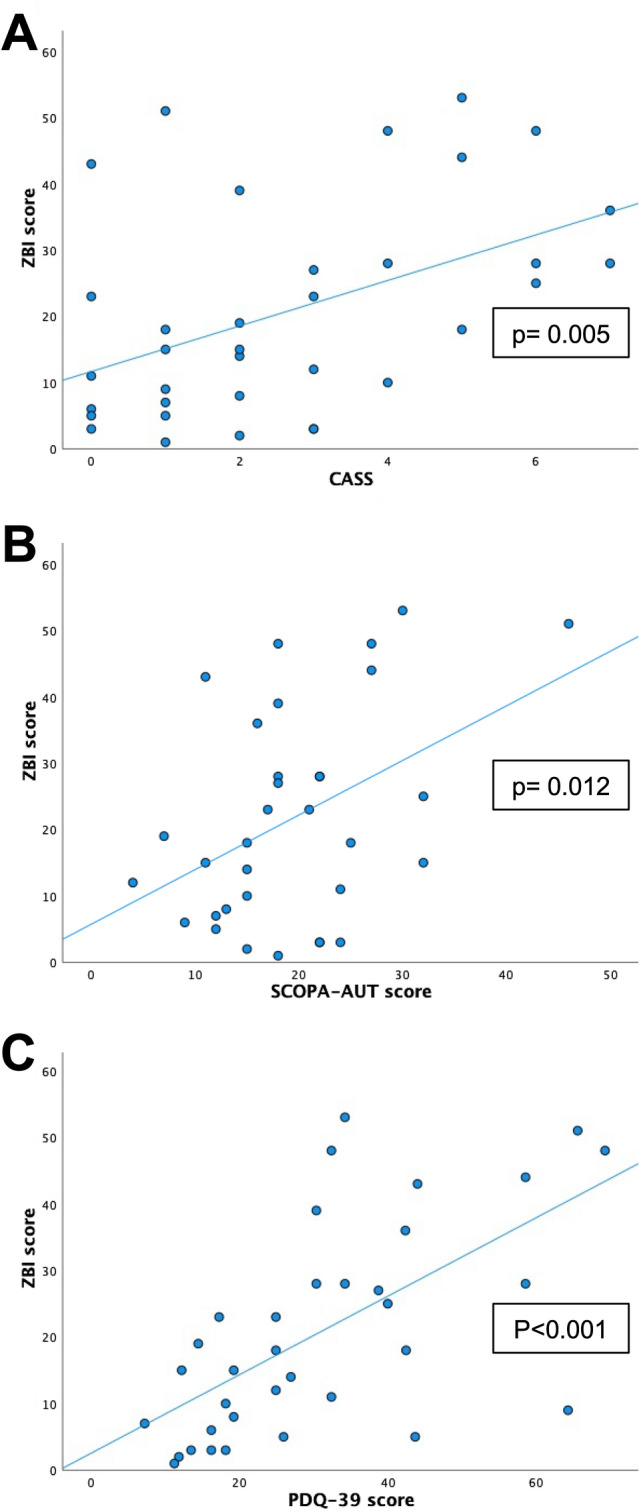
Correlation between higher caregiver burden, cAN severity, and patient quality of life. The caregiver burden, as measured by means of the ZBI score, is associated with cAN severity (**A**, **B**) and with health-related quality of life of patient (**C**). *CASS* composite autonomic system score, *PDQ-39* 39-item Parkinson’s Disease Questionnaire single index, *SCOPA-AUT* Scales for Outcomes in Parkinson’s Disease-Autonomic questionnaire, *ZBI* Zarit Burden Interview

There was a direct correlation between caregiver burden (ZBI score) and: (a) cAN severity (CASS) (*β* = 0.466; *p* = 0.005; Fig. 2A); (b) global autonomic impairment severity (SCOPA-AUT score) (*β* = 0.432; *p* = 0.012; Fig. 2B, C) patient’s HRQoL impairment (PDQ-39, single index) (*β* = 0.627; *p* < 0.001; Fig. 2C), even after correcting for patient’s age, disease duration, cognitive status, and motor disability, as well as caregiver’s age.

## Discussion

The main finding of the current study was that caregivers of patients with PD affected by cAN reported tenfold higher odds of moderate-severe burden, even after correcting for potential confounders, such as patients’ and caregivers’ age, PD duration, cognitive impairment, and motor disability. The ZBI score was associated with higher CASS, SCOPA-AUT, and PDQ-39 scores, showing a direct correlation between caregivers’ burden, patient autonomic impairment, and worse HRQoL.

Autonomic dysfunction may affect patients with PD at any clinical stage, with a prevalence that increases with disease duration [2, 25]. Previous studies showed an association between cAN and lower HRQoL, greater health care needs, and financial burden [2, 9, 25], as well as higher risk of PD complications, worse ADL impairment, and dementia [2, 4–6, 25]. Still, several aspects remain to be clarified about the association between cAN and burden of care in PD. A previous study investigated the impact of neurogenic OH on HRQoL [6], without addressing the issue of the impact that OH poses on the caregiver’s burden. Another study dealt with the caregiver burden and found a moderate correlation with general dysautonomia (SCOPA-AUT score) [12]. However, it did not focus on the cardiovascular component of dysautonomia, which is one of the most disabling autonomic features due to orthostatic symptoms and increased risk of falls.

PD has a major social and economic impact on caregivers’ life, which frequently causes early retirement from social and working activities [26]. It has been demonstrated that caring for a person with PD may cause an increased risk of psychiatric comorbidities and persistent distress [27]. PD duration, falls, cognitive impairment, hallucinations, and depressive symptoms represent the main predictors for a more severe caregiver strain [27]. Moreover, nonmotor symptoms of PD play a prominent role in determining greater caregiver burden [28]. Our study confirms these data and highlights the independent role of cardiovascular dysautonomia in increasing the caregiver burden, as reflected by the direct correlation between ZBI scores and cAN severity. No differences were found in ZBI scores, both in PDcAN^−^ and in PDcAN^+^ groups, between caregivers of patients reporting and not reporting falls; whether this observation is due to our small sample size or indicates a prominent role for cAN in determining greater caregiver burden still needs to be clarified.

In our sample, caregivers’ gender was equally distributed in both PDcAN^+^ and PDcAN^−^ group, mitigating possible gender-related differences in caregiver burden. Some data available in previous literature suggest a role for female gender in determining higher caregiver burden [29–31]; however, we did not find any significant difference in burden between male and female caregivers.

Importantly, we found a strong association between caregiver burden and patients’ HRQoL. This might suggest that caregiver distress eventually reflects on patient quality of life, potentially affecting the well-being of both [32, 33]. Taken together, these data further reinforce the notion that identifying treatable causes of caregiver distress is a key unmet priority in PD.

The strengths of our study include (a) a precise characterization of cAN by autonomic neurophysiological testing; (b) the use of a validated scale, the ZBI, to evaluate caregiver burden; and (c) the control of potential confounders that may influence the caregiver’s burden, such as age, PD duration, cognitive impairment, and motor disability.

This study suffers from some limitations. In particular, (a) the single-center design and the relatively small sample size; (b) the lack of comorbidity data for patients and caregivers; and (c) the lack of caregiver psychological and cognitive evaluations.

These limitations notwithstanding, this is, to the best of our knowledge, the first study quantifying the caregiver burden in relation with the presence of cAN in patients with PD. Our observations underline the importance of this frequent and potentially treatable complication of PD as a significant determinant of caregiver burden, which is a fundamental prerequisite for targeted psychosocial and economic interventions aiming at preventing the burnout of caregivers. Finally, effective caregiver support could reflect in healthcare cost savings. This proves relevant when considering that informal caregivers contribute to an annual spending savings of 375 billion USD in the USA only [33].

## Data Availability

The data that support the findings of this study are available from the corresponding author upon reasonable request.
